# Early diuretic use and mortality in critically ill patients with vasopressor support: a propensity score-matching analysis

**DOI:** 10.1186/s13054-019-2309-9

**Published:** 2019-01-10

**Authors:** Yanfei Shen, Weimin Zhang, Yong Shen

**Affiliations:** 1 Department of Intensive Care Unit, Zhejiang Hospital, No. 12, Lingyin Road, Hangzhou, Zhejiang 322100 People’s Republic of China; 2 Department of Intensive Care Unit, Dongyang People’s Hospital, No. 60, Wuning West Road, Dongyang, Zhejiang 322100 People’s Republic of China; 3 Department of Breast Surgery, The First Affiliated Hospital, Zhejiang Chinese Medical University, Hangzhou,, 310014 China

**Keywords:** Critical care, Loop diuretics, Vasopressor, Mortality, Fluid balance

## Abstract

**Background:**

The effect of loop diuretic use in critically ill patients on vasopressor support or in shock is unclear. This study aimed to explore the relationship between loop diuretic use and hospital mortality in critically ill patients with vasopressor support.

**Methods:**

Data were extracted from the Medical Information Mart for Intensive Care III database. Adult patients with records of vasopressor use within 48 h after intensive care unit admission were screened. Multivariable logistic regression and propensity score matching was used to investigate any association.

**Results:**

Data on 7828 patients were included. The crude hospital mortality was significantly lower in patients with diuretic use (166/1469 vs. 1171/6359, *p* <  0.001). In the extended multivariable logistic models, the odds ratio (OR) of diuretic use was consistently significant in all six models (OR range 0.56–0.75, *p* < 0.05 for all). In the subgroup analysis, an interaction effect was detected between diuretic use and fluid balance (FB). In the positive FB subgroup, diuretic use was significantly associated with decreased mortality (OR 0.64, 95% confidence interval (CI) 0.51–0.78) but was insignificant in the negative FB subgroup. In the other subgroups of mean arterial pressure, maximum sequential organ failure assessment score, and lactate level, the association between diuretic use and mortality remained significant and no interaction was detected. After propensity score matching, 1463 cases from each group were well matched. The mortality remained significantly lower in the diuretic use group (165/1463 vs. 231/1463, *p* < 0.001).

**Conclusions:**

Although residual confounding cannot be excluded, loop diuretic use is associated with lower mortality.

**Electronic supplementary material:**

The online version of this article (10.1186/s13054-019-2309-9) contains supplementary material, which is available to authorized users.

## Background

Optimizing fluid status is fundamental in critical care but is challenging to achieve. Sufficient fluid resuscitation is crucial in stabilizing the hemodynamic status of patients, and largely depends on large-volume fluid administration. Additionally, a positive correlation between fluid overload and adverse outcomes has been reported in various diseases, such as sepsis [1, 2], acute lung injury [3], and acute kidney injury (AKI) [4].

Loop diuretics are commonly prescribed to alleviate fluid overload. However, their efficacy and safety remain unclear. Several studies have shown that diuretics may induce oxidative stress and further aggravate renal injury in patients with severe AKI [5, 6]. However, a pilot randomized controlled trial (RCT) failed to confirm any specific incremental risk with furosemide use in AKI patients [7]. Furthermore, Grams et al. found that diuretic therapy was associated with improved survival in AKI patients [8]. Despite these contradictory conclusions, loop diuretics are still widely used in clinical practice to increase urine output (UO) and reduce the rate of edema. In a large survey of critically ill patients, diuretics were prescribed in more than 50% of the population during intensive care unit (ICU) stay [9]. Another large online questionnaire survey also reported that furosemide was commonly used, especially in critically ill patients with positive fluid balance (FB) and acute pulmonary edema [10]. In clinical situations, a patient’s hemodynamic status may become relatively stable with vasopressor support after adequate fluid resuscitation. One major concern is that diuretic use may induce hypovolemia and further compromise the hemodynamic status, and it remains unclear if diuretics can alleviate the detrimental effects of excessive fluid accumulation in these patients.

This study aimed to explore the relationship between diuretic use and hospital mortality in critically ill patients on vasopressor support, using logistic regression and propensity score matching (PSM). Subgroup analysis was also performed to investigate the interaction effect between diuretic use and other potential covariates.

## Methods

### Database introduction

All the data in the current study were extracted from an online international database— Medical Information Mart for Intensive Care III (MIMIC III)—that was published by the Massachusetts Institute of Technology, with approval from the review boards of the Massachusetts Institute of Technology and Beth Israel Deaconess Medical Center [11]. All the patients in the database were de-identified for privacy protection, and the need for informed consent was waived. One author (Y S) obtained access to this database (certification number 1564657) and was responsible for data extraction.

### Inclusion and exclusion criteria

Adult patients with medical records indicating any vasopressor use within 48 h after ICU admission were initially screened. The recorded vasopressors included dobutamine, dopamine, norepinephrine, epinephrine, vasopressin, and phenylephrine. Patients who were younger than 18 years or spent less than 48 h in the ICU were excluded. For patients who were admitted to the ICU more than once, only the first ICU stay was considered.

### Data extraction

Data on the demographic characteristics, comorbidities, laboratory outcomes, diuretic use, FB, mean arterial pressure (MAP), and disease severity score were extracted from the database. Diuretic use was defined as the use of any loop diuretic within 48 h after ICU admission, including furosemide, torasemide, and bumetanide. Vasopressor use was defined as the use of any vasopressor agent, including norepinephrine, epinephrine, dobutamine, dopamine, vasopressin, and phenylephrine, within 48 h after ICU admission.

### Stratification and outcome definition

Subgroup analysis was performed to explore the possible interaction between diuretic use and hemodynamic indices as well as disease severity. Stratification was performed according to the FB status within 48 h after ICU admission (≥ or < 0 ml/kg/48 h), mean MAP (≥ or < 70 mmHg), median value of maximum sequential organ failure assessment (SOFA) score during ICU stay (≥ or < 10), and median value of maximum lactate level during ICU stay (≥ or < 2.7 mmol/L). The primary endpoint was hospital mortality. AKI was defined as a 1.5-fold increase in serum creatinine (sCr) level during the ICU stay relative to baseline sCr level, according to the creatinine-based Kidney Disease Improving Global Outcomes criteria, without urine output [12, 13]. For patients without previous sCr data, it was estimated using the following formula [14]: sCr = 0.74 – 0.2 (if female) + 0.08 (if black) + 0.0039 × age (in years).

### Propensity score matching

PSM [15] was used to minimize the effect of confounding factors such as hemodynamic indices and disease severity, which may lead to outcome bias. The propensity score was assigned based on the probability that a patient would receive diuretic therapy and estimated using a multivariable logistic regression model. A one-to-one nearest neighbor matching algorithm was applied using a caliper width of 0.05. The following variables were selected to generate the propensity score: age, weight, diabetes mellitus, hypertension, cardiac disease, AKI, SOFA score on ICU admission, white blood cells, fluid intake volume, and proportions of different vasopressors. Kernel density plots of the *p* score were used to examine the PSM degree. Finally, 1463 matched pairs were generated and applied to further analyses.

### Management of missing data

Variables with missing data are common in the MIMIC III database. For serum lactate and albumin values, more than 20% were missing and were removed from this analysis. For other continuous variables with missing values less than 5%, the missing values were replaced by the mean or median values.

### Statistical analysis

Continuous variables are expressed as mean ± standard deviation or median (interquartile range), as appropriate. The student’s *t* test, analysis of variance, Wilcoxon rank-sum test, or Kruskal-Wallis test was used, as appropriate. Categorical data are expressed as proportions and compared using the χ^2^ test. An extended logistic model approach was used for covariate adjustment: as hemodynamic indices such as FB, MAP, and serum lactate level and disease severity are important factors affecting the decision to use diuretics in clinical practice, subgroup analyses were performed as described above. Multicollinearity was tested using the variance inflation factor (VIF) method, with a VIF ≥ 5 indicating the presence of multicollinearity. Goodness of fit tests were applied to all logistic regression models. PSM was used to minimize the imbalance between groups. A two-tailed test was performed, and *p* <  0.05 was considered statistically significant. All statistical analyses were performed using Stata 11.2 (Stata Corp., College Station, TX, USA).

## Results

### Baseline characteristics

**Fig. 1:**
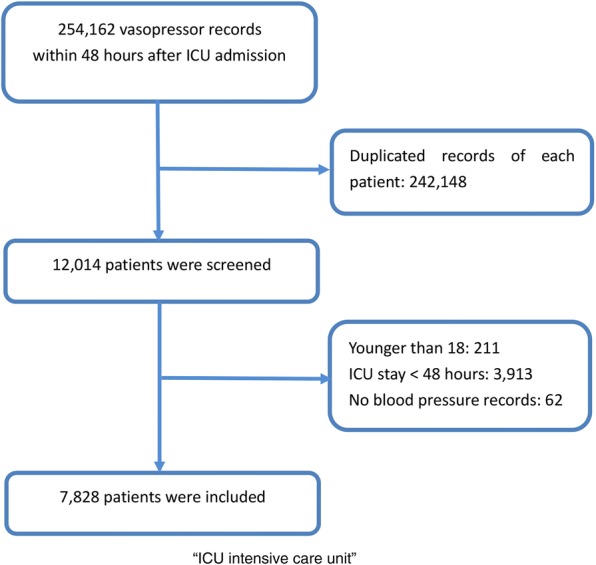
Flow chart of patient selection from the MIMIC III database

**Fig. 2:**
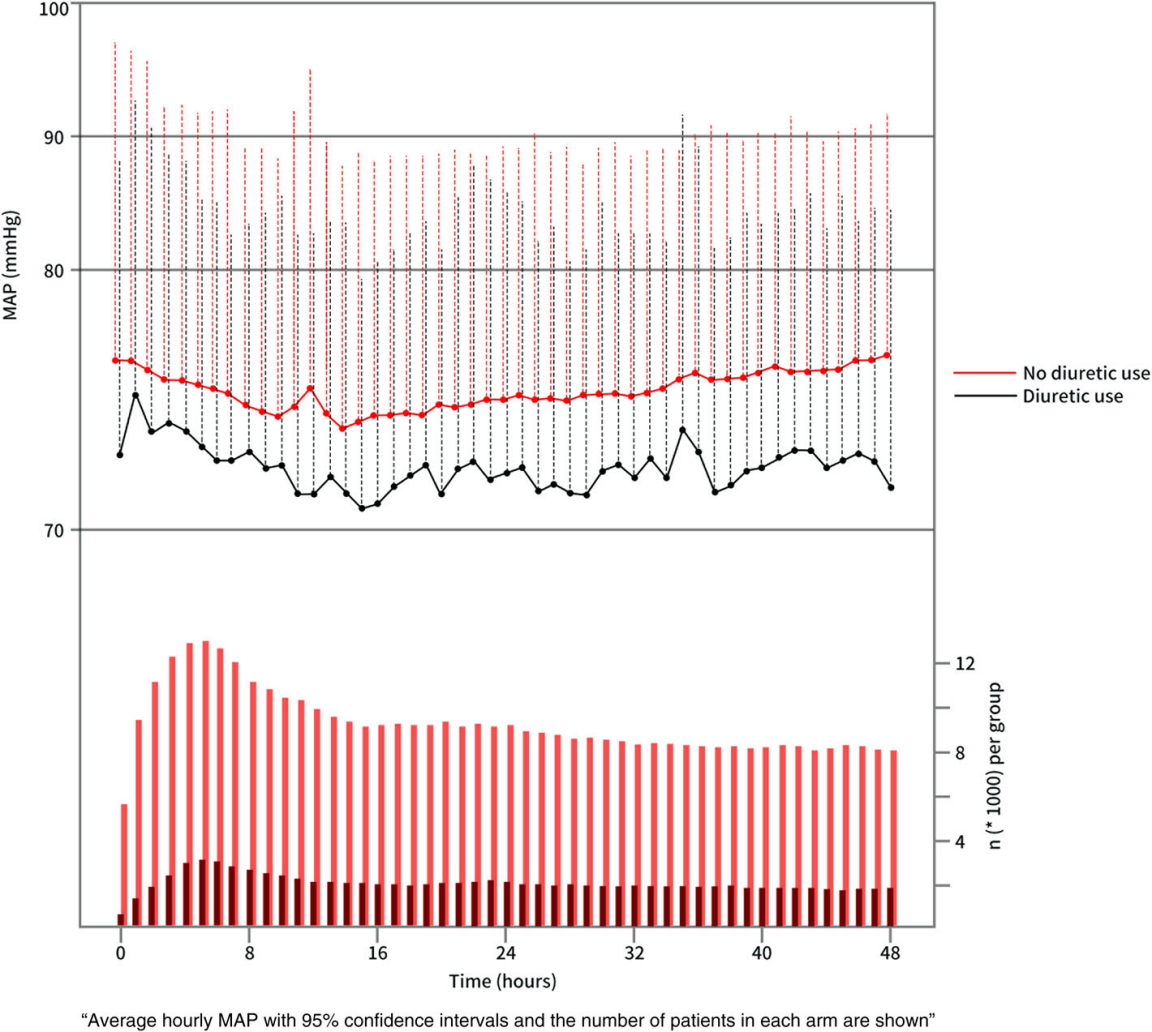
Trends of the average hourly mean arterial pressure (MAP) in patients with or without diuretic use within 48 h after ICU admission

Data on 7828 patients were included. The flow chart of patient selection is presented in Fig. 1. The comparisons of the baseline characteristics are listed in Table 1. The overall hospital mortality rate was 17.1%. The difference in MAP was small between the groups (74.6 ± 8.2 vs. 73.0 ± 7.1, *p* < 0.001), and the trends of the average hourly MAP are presented in Fig. 2. The SOFA score on admission was also relatively similar in the diuretic use and no diuretic use groups (6 (4–8) vs. 6 (4–9), *p* = 0.012). The fluid intake within 48 h was significantly higher in patients with no diuretic use (113.8 ± 77.1 vs. 95.5 ± 55.6, *p* < 0.002) while the UO volume was similar across the groups despite statistical significance (48.5 ± 38.2 vs. 51.2 ± 28.4, *p* = 0.009). However, the hospital mortality was significantly lower in patients with diuretic use (166/1469 vs. 1171/6359, *p* < 0.001). Additional baseline information, including the doses and proportions of different vasopressors, is presented in Additional file 1 (Table S1).

**Table 1:** Comparisons of the baseline characteristics between patients with and without diuretic use

Variables	All patients (*n* = 7828)	No diuretic use (*n* = 6359)	Diuretic use (*n* = 1469)	*P*
Age (years)	67.0 ± 14.2	66.5 ± 14.5	70.0 ± 12.3	< 0.001
Male, *n* (%)	4588 (58.6)	3749 (58.9)	839 (57.1)	0.196
Weight (kg)	82.3 ± 22.6	81.7 ± 22.3	84.9 ± 24.0	< 0.001
Emergency, *n* (%)	6072 (77.5)	5075 (79.8)	998 (67.9)	< 0.001
Comorbidities, *n* (%)				
Diabetes mellitus	2390 (30.5)	1850 (29.1)	540 (36.7)	< 0.001
Hypertension	3831 (48.9)	3025 (47.5)	806 (54.8)	< 0.001
Cardiac disease	3431 (43.8)	2603 (41.2)	848 (57.7)	< 0.001
Acute or chronic heart failure	2986 (38.1)	2329 (36.6)	657 (44.7)	< 0.001
Intracranial hemorrhage	273 (3.5)	249 (3.9)	24 (1.6)	< 0.001
Fluid balance				
Fluid intake (ml/kg/48 h)	110.3 ± 73.9	113.8 ± 77.1	95.5 ± 55.6	< 0.001
Urine output (ml/kg/48 h)	49.0 ± 36.6	48.5 ± 38.2	51.2 ± 28.4	0.009
Fluid balance (ml/kg/48 h)	45.1 ± 76.7	49.9 ± 79.7	24.2 ± 57.9	< 0.001
MAP after ICU admission				
MAP on ICU admission (mmHg)	77.4 ± 17.3	77.7 ± 17.6	76.2 ± 15.8	0.002
Mean MAP (mmHg)	74.3 ± 8.1	74.6 ± 8.2	73.0 ± 7.1	< 0.001
Maximum MAP (mmHg)	115.6 ± 40.1	115.9 ± 38.7	114.4 ± 45.5	0.19
Minimum MAP (mmHg)	48.2 ± 14.7	48.3 ± 14.5	47.7 ± 15.3	0.139
Disease severity scores, median (IQR)				
SOFA score on ICU admission	6 (4–8)	6 (4–8)	6 (4–9)	0.0122
Maximum SOFA score during ICU stay	10 (8–13)	10 (8–13)	11 (8–13)	0.2377
SAPS II on ICU admission	41 (32–52)	41 (32–52)	41 (34–51)	0.0151
GCS score on ICU admission	7 (3–14)	8 (3–14)	4 (3–11)	< 0.001
Maximum GCS score during ICU stay	15 (15–15)	15 (15–15)	15 (15–15)	0.001
Biochemical indices				
Maximum serum creatinine level (mg/dl)	1.87 ± 1.75	1.88 ± 1.81	1.83 ± 1.45	0.254
Maximum white blood cell count (10^9^/l)	18.5 ± 11.5	18.7 ± 11.8	17.5 ± 9.9	< 0.001
Maximum serum lactate level (mmol/l)	3.7 ± 3.2 (*n* = 6168)	3.7 ± 3.4 (*n* = 4799)	3.5 ± 2.7 (*n* = 1369)	0.004
Oxygenation indexes				
Mechanical ventilation, *n* (%)	6646 (84.9)	5323 (83.7)	1323 (90.0)	< 0.001
Duration of mechanical ventilation (h)	98.2 ± 175.8	102.9 ± 181.8	77.8 ± 145.7	< 0.001
PO_2_/FiO_2_ at ICU admission	258.0 ± 137.3	264.1 ± 140.2	232.6 ± 212.3	< 0.001
Minimum PO_2_/FiO_2_ during ICU stay	156.2 ± 103.5	154.8 ± 104.5	161.8 ± 99.0	0.029
Clinical outcomes				
Hyponatremia, *n* (%)*	3100 (39.6)	2433 (38.2)	667 (45.4)	< 0.001
Hypokalemia, *n* (%)*	2981 (38.1)	2475 (38.9)	506 (34.4)	0.001
Hypocalcemia, *n* (%)*	5569 (71.1)	4608 (72.4)	961 (65.4)	< 0.001
ICU LOS (days)	7.5 ± 8.5	7.7 ± 8.7	6.7 ± 7.4	< 0.001
Hospital LOS (days)	13.7 ± 12.1	13.9 ± 12.6	12.5 ± 10.0	< 0.001
AKI, *n* (%)	2358 (30.1)	1848 (29.1)	510 (34.7)	< 0.001
AKI stage 3, *n* (%)	1108 (14.1)	917 (14.4)	191 (13.0)	0.160
Dialysis, *n* (%)	680 (8.7)	561 (8.8)	119 (8.1)	0.376
Hospital mortality, *n* (%)	1337 (17.1)	1171 (18.4)	166 (11.3)	< 0.001

Values are shown as mean ± standard deviation unless otherwise indicated

*AKI* acute kidney injury, *GCS* Glasgow Coma Scale, *ICU* intensive care unit, *IQR* interquartile range, *LOS* length of stay, *MAP* mean arterial pressure, *SAPS II* Simplified Acute Physiology Score II, *SOFA* sequential organ failure assessment

* These three electrolyte disturbances were diagnosed according to the electrolyte value within 48 h after ICU admission

### Association between diuretic use and hospital mortality

**Fig. 3:**
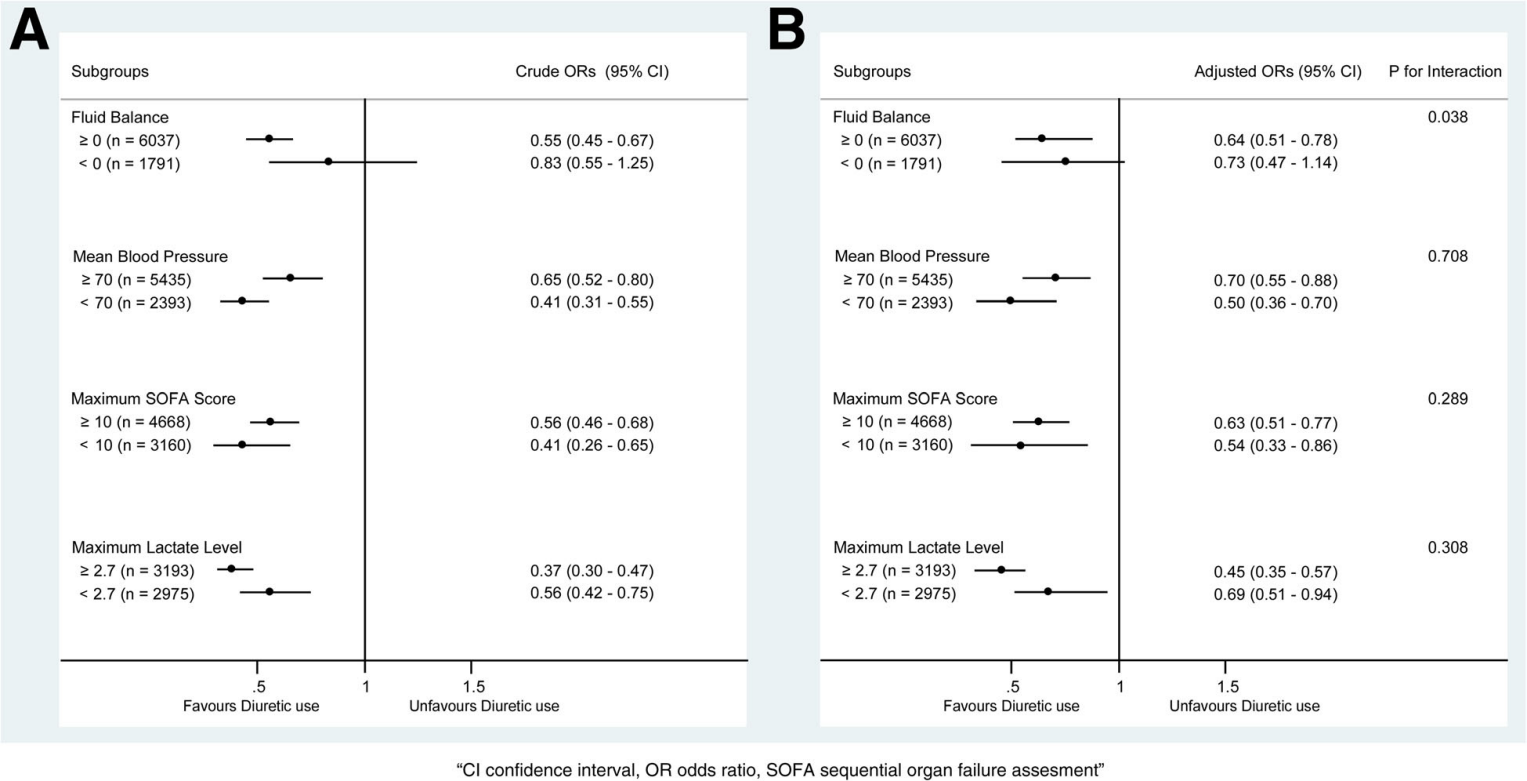
Subgroup analysis of the association between hospital mortality and diuretic use

In the extended multivariable logistic models (Table 2), we observed that the odds ratio (OR) of diuretic use was consistently significant in all six models (OR range 0.56–0.75, *p* < 0.05 for all). Subgroup analysis was performed according to the FB, MAP, maximum SOFA score, and lactate level (Fig. 3). The OR of diuretic use was significant in the MAP subgroups (≥ 70 mmHg: OR 0.70, 95% confidence interval (CI) 0.55–0.88, *p* = 0.002; < 70 mmHg: OR 0.50, 95% CI 0.36–0.70, *p* < 0.001), SOFA score (≥ 10: OR 0.63, 95% CI 0.51–0.77, *p* < 0.001; < 10: OR 0.54, 95% CI 0.33–0.86, *p* = 0.010), and lactate level (≥ 2.7: OR 0.45, 95% CI 0.35–0.57, *p* < 0.001; < 2.7: OR 0.69, 95% CI 0.51–0.94, *p* = 0.020), and no significant interaction was observed. However, in the FB subgroups, an interactive effect was detected (*p* value for interaction, 0.038). In the positive FB group, diuretic use was significantly associated with decreased mortality (OR 0.64, 95% CI 0.51–0.78, *p* < 0.001), and insignificantly associated with it in the negative FB group (OR 0.73, 95% CI 0.47–1.14, *p* = 0.170).

**Table 2:** Association between diuretic use and hospital mortality using an extended model approach

	Odds ratio of diuretic use	95% confidence interval	*P*
Model 1	0.56	(0.47–0.67)	< 0.001
Model 2	0.62	(0.51–0.74)	< 0.001
Model 3	0.58	(0.48–0.71)	< 0.001
Model 4	0.69	(0.55–0.81)	< 0.001
Model 5	0.75	(0.62–0.91)	0.004
Model 5	0.69	(0.57–0.84)	< 0.001

Adjusted covariates: Model 1 = diuretic use. Model 2 = Model 1 + (comorbidities including intracranial hemorrhage, hypertension, acute kidney injury, coronary disease and diabetes). Model 3 = Model 2 + (gender, age, biochemical indices including white blood cell count and SOFA score on ICU admission). Model 4 = Model 3 + (fluid balance within 48 h after intensive care unit admission). Model 5 = Model 4 + (proportion of patients receiving vasopressors including norepinephrine, epinephrine, dopamine, and dobutamine). Model 6 = Model 4 + (dose of vasopressors including norepinephrine, epinephrine, dopamine, and dobutamine)

Note: The mean variance inflation factor was 2.58 and 2.42 and the *p* value of the goodness of fit was 0.99 and 0.99 for Model 5 and Model 6, respectively

### Outcomes after propensity score matching

After PSM, 1463 cases from each group were well matched by a 1:1 matching algorithm (Table 3). The overall quality of the matched sample was assessed by comparing the standardized difference of the means and the ratio of the variances between the propensity scores of both groups as well as by graphically inspecting the propensity scores between the groups (Additional file 1: Figure S1). There was no significant difference between the two matched groups with regards to all twelve covariates, including fluid intake (94.1 ± 57.4 vs. 95.6 ± 55.7, *p* = 0.483) and proportions of different vasopressors. Among the 1463 propensity-matched pairs, we found that the hospital mortality was significantly lower in the diuretic use group (165/1463 vs. 231/1463, *p* < 0.001). The UO volume was significantly higher in the diuretic use group (51.3 ± 28.5 vs. 44.5 ± 28.6, *p* < 0.001), and the mean MAP was comparable (73.0 ± 7.1 vs. 74.5 ± 7.8, *p* < 0.001).

**Table 3:** Comparisons of the covariates after propensity score matching

Variables	No diuretic use (n = 1463)	Diuretic use (*n* = 1463)	*P*
Age (years)	70.3 ± 11.9	69.9 ± 12.3	0.472
Weight (kg)	84.4 ± 23.1	84.9 ± 24.0	0.599
Diabetes mellitus, *n* (%)	514 (35.2)	535 (36.5)	0.418
Hypertension, *n* (%)	820 (53.5)	802 (54.8)	0.503
Cardiac disease, *n* (%)	829 (58.5)	842 (57.5)	0.627
AKI, *n* (%)	496 (34.1)	504 (34.4)	0.755
SOFA score on ICU admission, median (IQR)	6 (4–8)	6 (4–9)	0.999
Maximum white blood cell count (10^9^/l)	17.0 ± 7.6	17.5 ± 9.9	0.198
Fluid intake (ml/kg/48 h)	94.1 ± 57.4	95.6 ± 55.7	0.483
Norepinephrine (mg/kg/48 h)	0.053 ± 0.139	0.052 ± 0.151	0.814
Dopamine (mg/kg/48 h)	0.782 ± 3.054	0.843 ± 3.665	0.623
Dobutamine (mg/kg/48 h)	0.193 ± 1.118	0.238 ± 1.789	0.411
Epinephrine (mg/kg/48 h)	0.008 ± 0.125	0.009 ± 0.084	0.676
Vasopressin (mg/kg/48 h)	0.071 ± 0.353	0.080 ± 0.359	0.495
Mechanical ventilation	1317	1317	1.000
Clinical outcomes			
Hospital mortality, *n* (%)	231 (15.6)	165 (11.2)	< 0.001
Urine output (ml/kg/48 h)	44.5 ± 28.6	51.3 ± 28.5	< 0.001
Fluid balance (ml/kg/48 h)	35.3 ± 53.8	24.3 ± 58.1	< 0.001
Mean MAP (mmHg)	74.5 ± 7.8	73.0 ± 7.1	< 0.001

Values are shown as mean ± standard deviation unless otherwise indicated

*AKI* acute kidney injury, *ICU* intensive care unit, *IQR* interquartile range, *MAP* mean arterial pressure, *SOFA* sequential organ failure assessment

### Sensitivity analysis

Vasopressin (despite weak recommendations in the sepsis guidelines) and phenylephrine are usually not used as first-line vasopressors. For example, some vasopressors may be used in patients during postoperative recovery from anesthesia, and vasopressin may be prescribed for gastrointestinal bleeding instead of septic shock. To test the robustness of our findings, we performed a sensitivity analysis excluding these two vasopressors. The outcomes remained stable after adjustment for confounders (Additional file 1: Table S2) and in the comparisons after PSM (Additional file 1: Table S3).

## Discussion

The present study demonstrates that in critically ill patients with vasopressor use the use of diuretics is associated with significantly decreased hospital mortality. This result was robust in the PSM analysis after adjustment for covariates and remained consistent in the subgroups of MAP, lactate level, and SOFA score. Unexpectedly, an interaction was detected between FB and diuretic use. A potential benefit was only observed in patients with positive FB; this benefit was insignificant in the negative FB subgroup. Our findings are suggestive of a possible beneficial role for diuretics in patients with vasopressor use, which has not been previously demonstrated.

Adequate fluid resuscitation is fundamental in critical care [16, 17], and entails aggressive large-volume fluid intake. However, several studies have reported that positive FB was strongly associated with worse outcomes [18], such as respiratory dysfunction [3], high intra-abdominal pressure [19], coagulation disorder [20], and increased mortality [21]. Excessive fluid accumulation may impair clinical outcomes. Several studies have demonstrated that strategies aimed at avoiding an excessive positive FB such as restricted fluid resuscitation may be beneficial. One systematic review found that restricted fluid resuscitation was associated with decreased mortality in trauma patients compared with initial liberal fluid resuscitation [22]. Silversides et al. also reported that in patients with acute respiratory distress syndrome or sepsis a conservative fluid strategy resulted in a shorter duration of ventilator use and ICU stay [23]. Thus, the use of strategies aimed at alleviating fluid overload following initial fluid resuscitation has become very important in ensuring better prognosis, suggesting the potential role of diuretic use. However, in contrast to the established knowledge on fluid accumulation, conclusions on the effectiveness and safety of loop diuretics in critically ill patients remain controversial.

In a pharmacoepidemiologic evaluation [24], furosemide was listed as a nephrotoxic drug. Mehta et al. investigated data from 552 AKI patients and reported that diuretic use was significantly associated with higher mortality and worse renal function [25]. In critically ill patients requiring acute dialysis post-surgery, a higher accumulative diuretic dose was significantly associated with increased mortality [26]. However, another multicenter, observational study reported that diuretics were commonly prescribed in 1743 patients with acute renal failure, and that their use was not associated with deteriorated outcomes, including mortality [27]. A small pilot RCT also failed to confirm any specific incremental risk with furosemide use in AKI patients, but suggested an insignificant downward trend in the 90-day mortality (21.6% vs. 30.5%) [7].

On reviewing all the aforementioned studies, we speculated that the risk of death or other poor outcomes was confounded by AKI severity (baseline sCr levels of 3.8 mg/dl [25], 3.3 mg/dl [26], 2.0 mg/dl [27], and 1.8 mg/dl [7]). Diuretic use in patients with severe AKI may contribute to unfavorable outcomes. Grams et al. concluded that post-AKI diuretic therapy was associated with improved 60-day survival in patients with relatively mild AKI (baseline sCr level 1.6 mg/dl) [8]. In our study, the mean baseline sCr level was 1.39 mg/dl and diuretic use was also associated with lower hospital mortality. Additionally, we speculated that the association between diuretic use and mortality might be mediated by fluid status. In the current study, the UO volumes in the two groups were similar and both reached the specific therapeutic end goals (UO *>* 1 ml/kg/h [28]) after adequate resuscitation. Meanwhile, the fluid intake volume was significantly higher in the no diuretic use group, which directly lead to a higher positive FB. In clinical practice, high fluid intake is one common strategy to reach resuscitation goals, including UO volume. However, due to the retrospective nature of the study, we could not infer if the UO volume impacted the fluid administration protocol decision. After adjustment for fluid intake using PSM, the FB was still significantly lower in the diuretic use group and largely depended on the increased UO; this was more significant in the sensitivity analysis. To a certain extent, this speculation also explained why this association was insignificant or even opposite in patients with severe AKI, as they may fail to respond to the diuretic challenge.

Furthermore, an interaction between FB and diuretic use was detected, and the benefit associated with diuretic use existed only in the positive FB subgroup; it remained insignificant in the negative FB subgroup. This finding further supports our speculation that in patients with a positive FB, the use of diuretics may alleviate fluid accumulation and thus contribute to improved survival. However, compared with achieving a mildly negative FB, achieving a greater degree of negative FB could not further reduce mortality in critically ill patients [29]. Thus, the use of loop diuretics may further increase the negative FB volume; however, this was not conducive to survival in the negative FB subgroup.

One major concern is that diuretic use may induce hypovolemia and compromise the already impaired hemodynamic status in such patients. In the current study, we found a significant difference in the MAP between the two groups, which could be affected by less fluid accumulation in the diuretic group. However, the clinical difference was relatively small both in the crude comparison and in the comparison after PSM (2–3 mmHg). This finding is consistent with the conclusion of Yeh et al. that furosemide in critically ill trauma patients resulted in lower fluid accumulation, with no detrimental effect on the hemodynamic parameters [30]. This finding was also supported by the results of a study by Shann [31]. Furthermore, we noticed that the association between diuretic use and hospital mortality was still significant in the subgroups with high SOFA scores, high maximum lactate levels, and low MAP, which suggested the feasibility of the appropriate use of loop diuretics in these patients.

This study has several limitations. First, there are many confounders for diuretic use in clinical practice, such as the reason for ICU admission, disease severity, kidney function, hemodynamic status, vasopressors, and clinician preference. In the current study, we included as many confounders as possible to minimize potential bias. However, due to the retrospective nature, some information was unavailable in this database. Rigorously designed randomized controlled studies may be the only solution for the imbalance between these two groups. Second, we inferred that the use of loop diuretics might reduce the mortality in this specific cohort. However, in addition to their diuretic effect, the adverse effects of loop diuretics, such as renal oxidative stress [5], may be overridden by the benefit of reduced positive FB. Caution should be exercised in interpreting these findings, especially in patients without fluid accumulation or those with severe AKI. Third, despite the benefit of vasopressors revealed in the current study, the inter-heterogeneity of included patients was still a concern. For instance, is the effect of diuretics consistent in patients with slight or massive vasopressor support? More studies are needed to verify this interaction. Fifth, missing data is an important issue in the analysis. In the current study, the percentage of missing values of most variables is relatively small. Thus, these missing values were replaced by their mean or median values instead of using multiple imputation methods. Finally, the causal relationship between diuretic use and mortality could not be confirmed. While the use of a propensity score can further support our speculation, it still cannot overcome the primary limitation associated with the observational nature of the study. Further prospective studies are needed to verify our hypothesis.

## Conclusions

Diuretic use was associated with reduced hospital mortality in critically ill patients with vasopressor support, without an obvious compromise in the MAP. This association was not affected by disease severity and serum lactate levels; however, this was only significant in patients with a positive FB. Future larger randomized clinical trials are required to confirm and validate this association.

## Additional file

Additional file 1: **Figure S1.** Kernel density plots of the propensity score before and after propensity score matching. **Table S1.** Comparisons of the baseline characteristics between patients with and without diuretic use. **Table S2.** Association between diuretic use and hospital mortality using an extended model approach (*n* = 4747). **Table S3.** Comparisons of covariates after propensity score matching. **Table S4.** Comparisons between subgroups with positive and negative fluid balance within 48 h after ICU admission. (DOCX 273 kb)

## Abbreviations

AKI: Acute kidney injury
CI: Confidence interval
FB: Fluid balance
ICU: Intensive care unit
MAP: Mean arterial pressure
MIMIC III: Medical Information Mart for Intensive Care III
OR: Odds ratio
PSM: Propensity score matching
RCT: Randomized controlled trial
sCr: Serum creatinine
SOFA: Sequential organ failure assessment
UO: Urine output
VIF: Variance inflation factor
