# Supplementary material for: Early diuretic use and mortality in critically ill patients with vasopressor support: a propensity score-matching analysis
Source: Crit Care. 2019 Jan 10;23:9. doi: 10.1186/s13054-019-2309-9 (PMC6329160; doi:10.1186/s13054-019-2309-9)
Supplement: Supplementary file 1 — Figure S1. Kernel density plots of the propensity score before and after propensity score matching. Table S1. Comparisons of the baseline characteristics between patients with and without diuretic use. Table S2. Association between diuretic use and hospital mortality using an extended model approach (n = 4747). Table S3. Comparisons of covariates after propensity score matching. Table S4. Comparisons between subgroups with positive and negative fluid balance within 48 h after ICU admission. (DOCX 273 kb) [file 13054_2019_2309_MOESM1_ESM.docx]

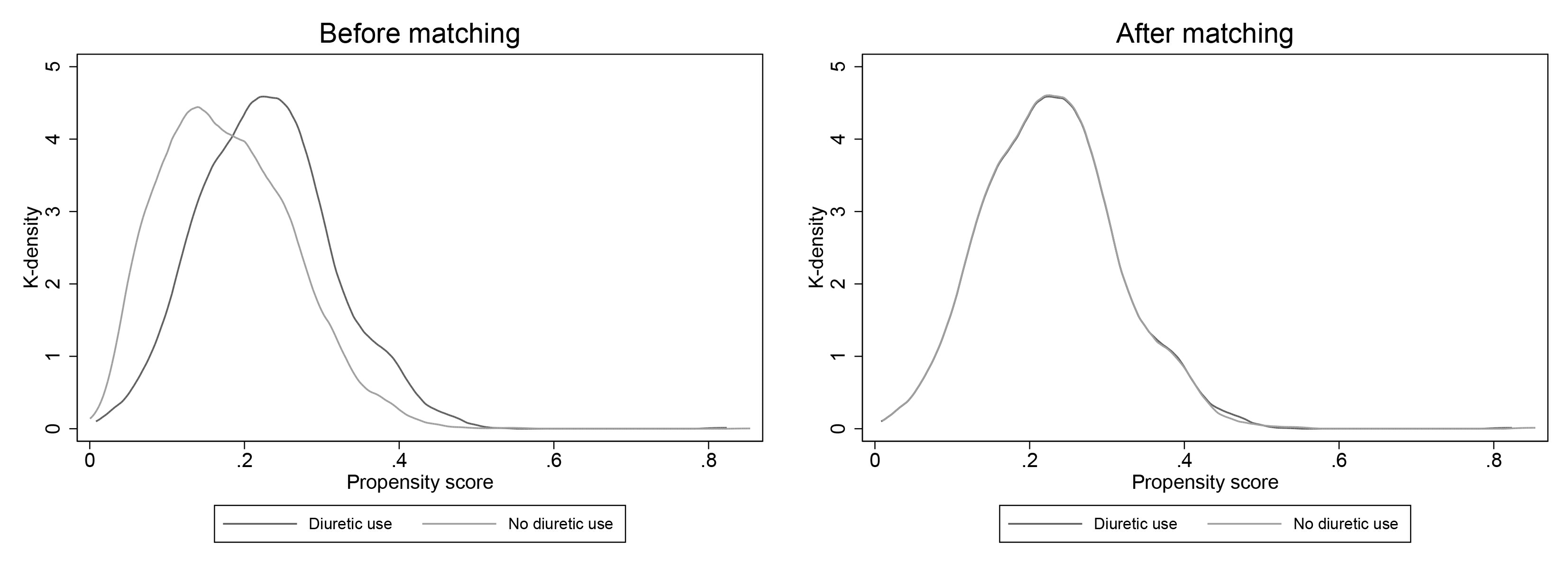


Figure S1 Kernel density plots of the propensity score before and after propensity score matching

Table S1 Comparisons of the baseline characteristics between patients with and without diuretic use

| Variables | All patients  (n = 7828) | No diuretic use  (n = 6359) | Diuretic use  (n = 1469) | p | |
| --- | --- | --- | --- | --- | --- |
| ICU type | | | | | |
| MICU [n (%)] | 1953 (24.9) | 1748 (27.5) | 205 (13.9) | | < 0.001 |
| CCU/CSRU [n (%)] | 4345 (55.5) | 3251 (51.1) | 1094 (74.5) | | < 0.001 |
| TSICU/SICU [n (%)] | 1530 (19.5) | 1360 (21.4) | 170 (11.5) | | < 0.001 |
| Ethnicity | | | | | |
| White [n (%)] | 5729 (73.2) | 4572(71.8) | 1157 (78.7) | | < 0.001 |
| Black [n (%)] | 391 (5.0) | 329 (5.2) | 62 (4.2) | | 0.131 |
| Asian [n (%)] | 179 (2.3) | 152 (2.4) | 27 (1.8) | | 0.202 |
| Biochemical indices |  |  |  | |  |
| Serum creatinine level on ICU admission (mg/dL) | 1.39 ± 1.36 | 1.43 ± 1.43 | 1.24 ± 0.97 | | < 0.001 |
| Maximum serum urea level (mg/dL) | 39.1 ± 29.1 | 38.8 ± 29.1 | 40.6 ± 29.4 | | 0.032 |
| Serum calcium level on ICU admission (mg/dL) | 8.09 ± 0.96 | 8.05 ± 0.98 | 8.27 ± 0.81 | | < 0.001 |
| Maximum serum sodium level (mmol/L) | 137.5 ± 4.8 | 137.7 ± 4.9 | 136.6 ± 4.0 | | < 0.001 |
| White blood cell count on ICU admission (10^9/L) | 13.9 ± 9.5 | 14.0 ± 10.0 | 13.2 ± 6.3 | | 0.002 |
| Hemoglobin level on ICU admission (g/dl) | 10.5 ± 2.4 | 10.6 ± 2.4 | 10.0 ± 2.2 | | < 0.001 |
| Platelet count on ICU admission (10^9/L) | 201.8 ± 109.3 | 204.9 ± 113.1 | 188.7 ± 89.9 | | < 0.001 |
| Serum lactate level on ICU admission (mmol/L) | 2.7 ± 2.1 (n = 6168) | 2.7 ± 2.2 (n = 4799) | 2.5 ± 1.8 (n = 1369) | | < 0.001 |
| Serum albumin level on ICU admission (g/dL) | 2.76 ± 0.64 (n = 4026) | 2.72 ± 0.63 (n = 3400) | 2.99 ± 0.65 (n = 626) | | < 0.001 |
| Time interval between ICU admission and first diuretic use (hours) | 4.5 ± 10.8 | 0 ± 0 | 24.2 ± 12.9 | | < 0.001 |
| Dose and proportion of different vasopressors |  |  |  | |  |
| Norepinephrine (mg/kg/48hours) | 0.079 ± 0.206 | 0.085 ± 0.216 | 0.051 ± 0.151 | | < 0.001 |
| Epinephrine (mg/kg/48hours) | 0.006 ± 0.068 | 0.005 ± 0.064 | 0.010 ± 0.084 | | 0.012 |
| Dobutamine (mg/kg/48hours) | 0.28 ± 1.93 | 0.30 ± 1.96 | 0.23 ± 1.78 | | 0.262 |
| Dopamine (mg/kg/48hours) | 1.14 ± 4.35 | 1.21 ± 4.49 | 0.84 ± 3.65 | | 0.003 |
| Vasopressin (mg/kg/48hours) | 0.086 ± 0.360 | 0.087 ± 0.360 | 0.080 ± 0.358 | | 0.486 |
| Phenylephrine (mg/kg/48hours) | 0.76 ± 1.60 | 0.79 ± 1.66 | 0.64 ± 1.28 | | < 0.001 |
| Norepinephrine [n (%)] | 3111 (39.7) | 2634 (41.4) | 477 (32.4) | | < 0.001 |
| Epinephrine [n (%)] | 1003 (12.8) | 696 (10.9) | 307 (20.8) | | < 0.001 |
| Dobutamine [n (%)] | 426 (5.4) | 363 (5.7) | 63 (4.3) | | 0.031 |
| Dopamine [n (%)] | 1327 (16.9) | 1136 (17.8) | 191 (13.0) | | < 0.001 |
| Vasopressin [n (%)] | 779 (9.9) | 650 (10.2) | 129 (8.7) | | 0.097 |
| Phenylephrine [n (%)] | 4818 (61.3) | 3759 (59.1) | 1059 (72.1) | | < 0.001 |

^Abbreviations: ICU, intensive care unit; MICU, medical intensive care unit; CCU, coronary care unit;^ ^CSRU, cardiac surgery recovery unit; SICU,^ ^surgical intensive care unit;TSICU, trauma surgical intensive care unit.^

^Note: Only vasopressors used within 48 hours were analyzed. Data on the dose of all the vasopressors were non-normally distributed. However, if using median (inter-quartile range), most of the data would be presented as 0 (0 - 0). For interpretation, all the data were presented as mean ± standard deviation.^

|  | Odds ratio of diuretic use | 95% confidence Interval | P value |
| --- | --- | --- | --- |
| Model 1 | 0.62 | (0.50 – 0.76) | < 0.001 |
| Model 2 | 0.67 | (0.53 – 0.83) | < 0.001 |
| Model 3 | 0.65 | (0.52–0.81) | < 0.001 |
| Model 4 | 0.74 | (0.59 – 0.92) | 0.009 |

Table S2 Association between diuretic use and hospital mortality using an extended model approach (n = 4747)

^Adjusted covariates: Model 1 = diuretic use. Model 2 = Model 1 + (comorbidities including intracranial hemorrhage, hypertension, acute kidney injury and coronary diseases). Model 3 = Model 2 + (biochemical indices including white blood cell count and SOFA score on ICU admission). Model 4 = Model 3 + (vasopressors including norepinephrine, epinephrine, dopamine and dobutamine).^

^Note: For Model 4, the mean variance inflation factor was 2.45 and^ ^the p value of the goodness of fit was 0.998.^

Table S3 Comparisons of covariates after propensity score matching

| Variable | No diuretic-use  (n = 795) | Diuretic-use  (n = 795) | p |
| --- | --- | --- | --- |
| Age (years) | 70.0 ± 12.2 | 70.4 ± 12.2 | 0.520 |
| Weight (kg) | 84.9 ± 23.8 | 85.6 ± 24.2 | 0.525 |
| Diabetes mellitus [n (%)] | 301 (37.8) | 308 (38.7) | 0.718 |
| Hypertension [n (%)] | 415 (52.2) | 410 (51.5) | 0.802 |
| Cardiac disease [n (%)] | 439 (55.2) | 438 (55.0) | 0.960 |
| AKI [n (%)] | 357 (39.8) | 339 (42.6) | 0.363 |
| SOFA on ICU admission  median (IQR) | 8 (6 – 10) | 8 (6 – 10) | 0.956 |
| Maximum white blood cell (10^9/L) | 18.3 ± 8.5 | 18.2 ± 7.7 | 0.806 |
| Fluid intake (ml/kg/48hrs) | 98.9 ± 62.2 | 98.7 ± 63.6 | 0.940 |
| Norepinephrine (mg/kg/48hours) | 0.097 ± 0.171 | 0.095 ± 0.195 | 0.855 |
| Dopamine (mg/kg/48hours) | 1.36 ± 4.31 | 1.54 ± 4.86 | 0.426 |
| Dobutamine (mg/kg/48hours) | 0.39 ± 2.29 | 0.43 ± 2.40 | 0.722 |
| Clinical outcomes |  |  |  |
| Hospital mortality [n (%)] | 165 (20.7) | 125 (15.7) | 0.009 |
| Urine output (ml/kg/48hr) | 40.7 ± 31.0 | 50.3 ± 29.6 | < 0.001 |
| Fluid balance (ml/kg/48hr) | 44.4 ± 68.8 | 29.6 ± 68.2 | < 0.001 |
| Mean MAP (mmHg) | 73.3 ± 7.7 | 72.5 ± 7.3 | 0.038 |

^Abbreviations: AKI, acute kidney injury; SOFA, sequential organ failure assessment; ICU intensive care unit; MAP mean arterial pressure.^

^Note: This is a sensitivity analysis performed by excluding vasopressin and phenylephrine.^

Table S4 Comparisons between subgroups with positive and negative fluid balance within 48 hours after ICU admission

| Variable | Positive fluid balance  (n = 6037) | Negative fluid balance  (n = 1791) | p |
| --- | --- | --- | --- |
| Dobutamine (mg/kg/48hours) | 0.287 ± 1.957 | 0.291 ± 1.861 | 0.943 |
| Dopamine (mg/kg/48hours) | 0.162 ± 4.473 | 1.072 ± 3.942 | 0.427 |
| Epinephrine (mg/kg/48hours) | 0.007 ± 0.078 | 0.002 ± 0.123 | 0.010 |
| Norepinephrine (mg/kg/48hours) | 0.094 ± 0.227 | 0.026 ± 0.090 | < 0.001 |
| Vasopressin (mg/kg/48hours) | 0.104 ± 0.397 | 0.023 ± 0.170 | < 0.001 |
| Phenylephrine (mg/kg/48hours) | 0.831 ± 1.718 | 0.558 ± 1.099 | < 0.001 |
| Dobutamine [n (%)] | 321 (5.3) | 105 (5.8) | 0.372 |
| Dopamine [n (%)] | 982 (16.2) | 345 (19.2) | 0.003 |
| Epinephrine [n (%)] | 814 (13.4) | 189 (10.5) | 0.001 |
| Norepinephrine [n (%)] | 2674 (44.3) | 436 (24.3) | < 0.001 |
| Vasopressin [n (%)] | 712 (11.7) | 67 (3.7) | < 0.001 |
| Phenylephrine [n (%)] | 3714 (61.5) | 1104 (61.6) | 0.962 |
| AKI [n (%)] | 2013 (33.3) | 345 (19.2) | < 0.001 |
| Mechanical ventilation [n (%)] | 5201 (86.1) | 1445 (80.6) | < 0.001 |
| Fluid intake (ml/kg/48hrs) | 123.6 ± 76.6 | 65.8 ± 38.5 | < 0.001 |
| Urine output (ml/kg/48hr) | 42.7 ± 33.9 | 70.3 ± 37.2 | < 0.001 |
| Fluid balance (ml/kg/48hr) | 65.9 ± 67.1 | - 25.0 ± 64.7 | < 0.001 |
| Initial MAP (mmHg) | 76.9 ± 17.5 | 79.3 ± 16.5 | < 0.001 |
| Mean MAP (mmHg) | 73.8 ± 7.96 | 75.8 ± 8.4 | < 0.001 |

^Abbreviations: AKI, acute kidney injury; MAP mean arterial pressure.^
